# CMOS Compatible Hydrogen Sensor Using Platinum Gate and ALD–Aluminum Oxide

**DOI:** 10.3390/s24103020

**Published:** 2024-05-10

**Authors:** Adham Elshaer, Serge Ecoffey, Abdelatif Jaouad, Stephane Monfray, Dominique Drouin

**Affiliations:** 1 Institut Interdisciplinaire d’Innovation Technologique (3IT), Université de Sherbrooke, 3000 Boul. Université, Sherbrooke, QC J1K 0A5, Canada; 2 Laboratoire Nanotechnologies Nanosystemes (LN2) CNRS IRL-3463, 3000 Boul. Université, Sherbrooke, QC J1K 0A5, Canada; 3 STMicroelectronics 850, rue Jean Monnet, 38926 Crolles, France

**Keywords:** hydrogen, sensing, CMOS compatible, MOS structure, ALD, platinum, fabrication, characterisation

## Abstract

In this study, a p-Si/ALD-Al_2_O_3_/Ti/Pt MOS (metal oxide semiconductor) device has been fabricated and used as a hydrogen sensor. The use of such a stack enables a reliable, industry-compatible CMOS fabrication process. ALD-Al_2_O_3_ has been chosen as it can be integrated into the back end of the line (BEOL) or in CMOS, post processing. The device response and recovery are demonstrated with good correlation between the capacitance variation and the hydrogen concentration. Detection down to 20 ppm at 140 °C was obtained and a response time of 56 s for 500 ppm was recorded.

## 1. Introduction

The ever-growing demand for alternative energy sources and carriers, such as hydrogen, in order to achieve carbon neutrality, requires major infrastructural investments and adaptation [1]. These adaptations include the development of a reliable hydrogen sensing technology (which adheres to safety standards) with less than 30 s response time and the ability to detect concentrations < 100 ppm [1,2,3]. Commercially available hydrogen sensors use different platforms, including electrochemicals, catalytic combustion, metal-oxide sensors (MOX), and field-effect-based sensors [4]. Semiconductor FET-based hydrogen sensors [5,6] provide many advantages: a fast response time, a long lifetime (compliant with standards [3]), compactness, and scalability [5]. Lundstrom et al. [7] were the first to demonstrate the use of a palladium-based MOS structure for hydrogen sensing applications in 1975. For such devices, hydrogen molecules dissociate at the catalytic metal surface, then hydrogen atoms diffuse through the metal to finally be adsorbed at the metal–insulator interface [8,9]. The polarised hydrogen atoms at the interface induce a dipole moment, which impacts the C-V characteristics of the MOS structure, adding a shift in ΔV.

∆V = (µ × ni)/ε0(1)

where µ is the dipole moment, ni is the concentration of hydrogen atoms at the interface, and ε0 is the dielectric permittivity of the vacuum. While ε0 is a constant, ni and µ can vary with either the choice of metal or the insulator [9,10,11,12,13,14]. Metals such as Pt, Pd, Ir, Rh, Pd/Pt, Pt/Pd, Rh/Pd, and Au/Pd [11,12,13,14] have all shown a C-V curve shift of the order of at least 110 mV for a concentration of 500 ppm [11,12,13,14]. MOS structures with a Pt/Pd metal gate show the highest shift of 350 mV [10]. Choi et al. have carried out a comparative study to show the long-term (>20 cycles) mechanical reliability and performance of different sensing MOS device fabricated using different gate materials; for more information refer to [14]. However, long-term performance varies according to the nature of the metal. Compared to other materials, Pd and Au/Pd showed a poor mechanical performance [14], characterised by the formation of blisters on the surface after repetitive exposure to hydrogen (>20x) [12,14]. Platinum (as a gate) or Pt/Pd were found to show a longer lifespan and better mechanical properties [12]. Despite these advantages, Pt’s adhesion to oxide represents a technological limitation, particularly for wafer-level fabrication [15]. A thin titanium layer could be deposited on the oxide to promote the adhesion of the catalytic Pt metal. However, the use of a Ti layer (e.g., 30 nm) can inhibit the MOS structure’s response to hydrogen [14]. This is probably the reason why only a few devices have used Ti/Pt as a sensing gate stack [14,16,17]. Usagawa et al. [17] demonstrated that post-metallisation annealing in air could be used to oxidize a thin, 5 nm Ti film and create TiO_x_. This approach was used to demonstrate the first Pt-TiO_x_-SiO_2_ MOSFET that can detect 100 ppm of hydrogen. While it is easy to overlook the importance of post-metallization annealing, it remains a key step; during this step, the 5 nm TiO_x_ film also diffuses through the Pt grain boundaries, further increasing the Pt–oxide interface and hence increasing the available number of hydrogen adsorption sites [18]. Moreover, e-beam-evaporated TiO_2_ has been studied in a Pd/TiO_2_/Si MOS structure [19,20]. Detection of a 10,000 ppm hydrogen concentration has been shown and an area-dependent drift in the MOS structure has been observed [19]. 

Multiple dielectrics can be employed to create a catalytic metal–insulator interface, e.g., SiO_2_ and Si_3_N_4_ [14,21]. While thermally grown SiO_2_ has been widely used in the fabrication of H_2_ MOS sensors, they are not BEOL-compatible [7], due to the high-temperature growth process. In this work, we propose the use of an atomic layer deposition (ALD) technique to deposit aluminium oxide (Al_2_O_3_) as a gate dielectric. This technique allows for a low processing temperature, high-quality dielectric films, and an Al_2_O_3_/Si interface with good electronic properties [7,16,17]. 

The use of a p-Si/Al_2_O_3_/Ti/Pt MOS device as a hydrogen sensor is demonstrated. The fabrication method, as well as the device’s performance under different testing conditions, is presented. The C-V curve drift after cyclic measurement was used to study the stability of the MOS device and the impact of different annealing conditions. A forming gas annealing process was found to be able to eliminate drift. Furthermore, the capacitance variation over time was used to monitor the sensor’s response and recovery at different hydrogen concentrations. The impact of operating temperature and total gas flow was investigated, showing much shorter response times at high temperatures and higher total gas flow.

## 2. Materials and Methods

MOS capacitors were fabricated using a p-Si wafer (1–5 Ω.cm). After an organic cleaning step, the standard RCA process was performed. Al_2_O_3_ was deposited by plasma-enhanced ALD at 200 °C; then, different post-oxide annealing (POA) conditions were tested in N_2_, O_2_, and H_2_/N_2_ atmospheres, all for the same duration of 30 min. Lithography was then carried out on individual samples, using a bright field mask and a negative-resisting AZnLOF 2020 for the lift-off process. A Pt/Ti 100 nm/5 nm gate metallisation material was deposited using e-beam evaporation. Finally, post-metallisation annealing at 400 °C was carried out for 24 h under N_2_. This 24 h PMA process at 400 °C is commonly used to make stable MOS hydrogen sensors [10,17]. 

**Figure 1:**
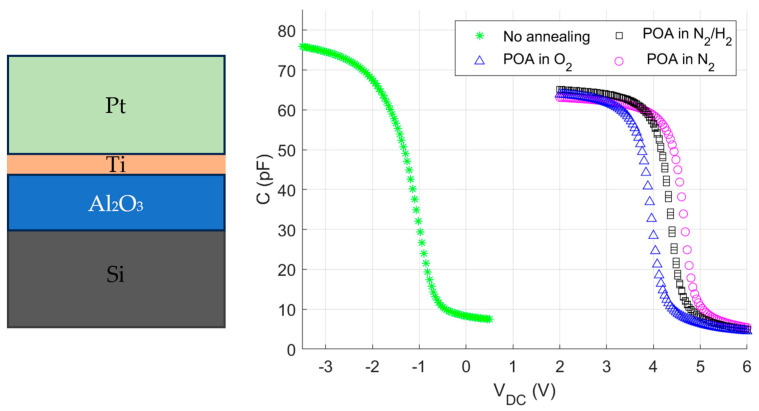
C-V characterization of the as-fabricated MOS devices at room temperature.

The as-fabricated MOS capacitors were characterised using a C-V measurement technique at 1 MHz at room temperature. As shown in Figure 1, all fabricated devices demonstrate the typical behaviour of an MOS device, with accumulation, depletion, and inversion regimes. It is important to note that, at RT, no drift was observed, even under multiple cycles of measurements.

It is worth noting that the annealing conditions have an impact on the position of the V_fb_ of the MOS capacitor along the *x*-axis. The non-annealed device shows V_fb_ at −1.1 V (green curve), while other devices show a V_fb_ between 4.4 V and 5.0 V.

The shift of the V_fb_ towards a positive voltage is associated with the presence of the negative, charge activated by annealing. This behaviour is well known for the ALD-Al_2_O_3_/Si interface and commonly used for field effect passivation [22]. ALD–aluminum oxide annealing in oxygen at 450 °C has been shown to modify the Al_2_O_3_–Si interface, in addition to forming Si-O bonds and modifying Al_2_O_3_ stoichiometry [23]. While N_2_ seems to impact the interface less, forming gas (H_2_/N_2_) yields the lowest interface trap (Dit) concentration; this will be shown later through C-V measurement at 140 °C. 

**Figure 2:**
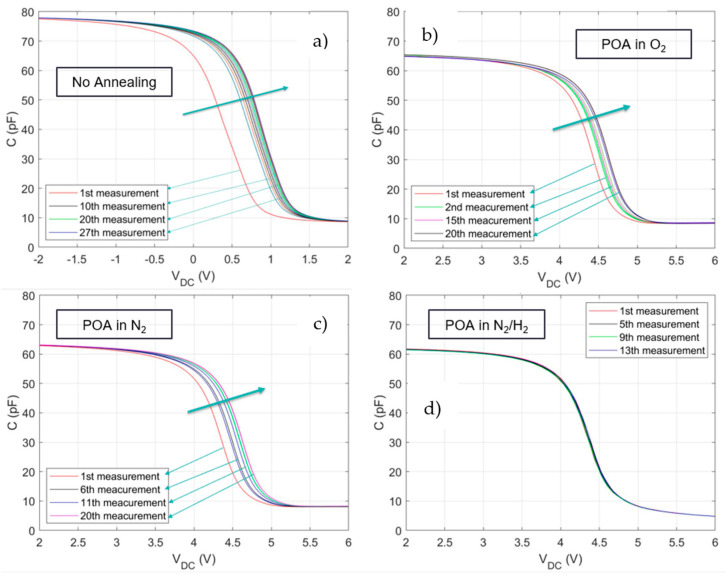
(**a**) C-V curve drift for a non-annealed device. (**b**) Results after O_2_ annealing. (**c**) Results after N_2_ annealing. (**d**) Results after H_2_/N_2_ annealing. All annealing steps were conducted at 450 °C for 30 min.

Since hydrogen MOS sensors are typically operated at approximately 140 °C [10,24], fabricated MOS capacitors are characterised at this temperature. Figure 2 includes the C-V characteristics of devices with different POA conditions. Without annealing (Figure 2a), after 27 measurement cycles, a stable C-V curve is obtained, with a corresponding accumulated V_fb_ drift of 0.52 V. Figure 2b,c show that, after annealing in O_2_ (2b) and N_2_ (2c), the accumulated curve drift is 0.24 V, stabilising after 20 cycles. After annealing to form gas, on the other hand (10% H_2_/N_2_), the drift at 140 °C was eliminated, as shown in Figure 2d. After 13 consecutive cycles of C-V measurements, the accumulated C-V curve drift is 0 V.

The stability observed upon annealing in H_2_/N_2_ could be attributed to the H_2_ passivation of non-terminated dangling bonds and free mobile ions in the ALD-Al_2_O_3_ oxide [22]. Furthermore, ALD-deposited Al_2_O_3_ has been found to be a highly effective hydrogen diffusion barrier [25]: at 680 °C, the maximum H_2_ diffusion has been experimentally shown to be 2.4 nm [25]; at 800 °C, it goes up to a maximum of 9.3 nm [25]. The C-V characteristics shown in Figure 2 are for MOS structures with 38 nm Al_2_O_3_; this prevents the permeation of hydrogen to the oxide/silicon interface and prevents permanent C-V curve drift upon exposure to H_2_. The thickness of Al_2_O_3_ was measured using ellipsometry showing a 38 nm thick film, with a refractive index (RI) of 1.6 at 623 nm wavelength. The RI value is close to values reported in the literature [26].

The measure thickness, along with the measured oxide capacitance, have been used to calculate the materials dielectric constant as per the following equation:

(2) $$\varepsilon_{r}=\frac{C_{ox}t_{ox}}{\varepsilon_{0}\times A}$$

The calculation yields $\varepsilon_{r}$ = 8.7. Additional measurements and structural characterization could provide further information on the Pt–insulator interface; examples would include surface roughness using atomic force microscopy (AFM), chemical composition, and comparative imaging of the interface before and after annealing. However, within the scope of this study, the main aim is to perform the electrical characterization of the MOS structure under H_2_ and the demonstration and evaluation of a reversible sensing response. For future device and more specifically interface optimization, structural analysis would be essential and shall be made.

**Figure 3:**
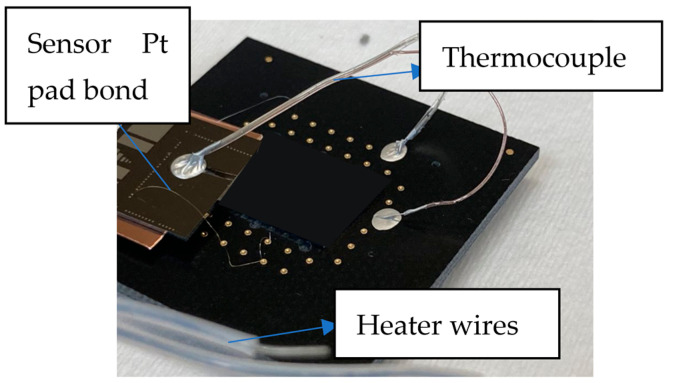
MOS capacitor wire bonded onto the PGA ready for characterization inside of hydrogen testing chamber at 140 °C.

The back ohmic contact, In/Ga on the backside of the silicon substrate is attached to a copper sheet using silver paste. The back-ohmic contact was attached to a copper sheet using silver paste. The entire assembly was mounted onto a heater using thermally conductive epoxy as shown in Figure 3. The heater was, itself, mounted on a pin grid array (PGA). Using gold ball bonding, the gate and the back-side contact of the 180 × 180 μm^2^ MOS-capacitor were connected to the socket pins. 

**Figure 4:**
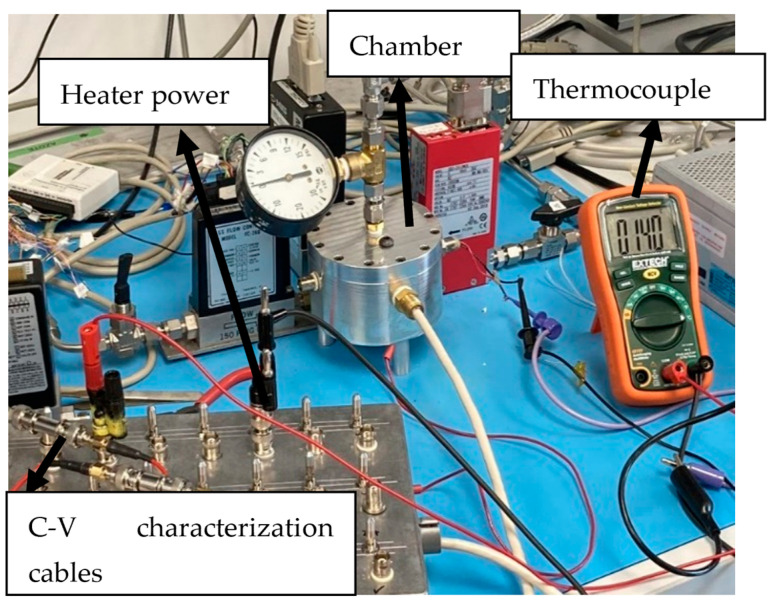
Test setup: two terminals for C-V characterization, two terminals for heater power supply, and two terminals for thermocouple temperature reading.

The testing setup shown in Figure 4 consists of a cylindrical aluminum chamber with a pressure gauge at the top exhaust port, mass flow controllers, and multiple sealed electrical ports for C-V characterization (SMA cables, heater and thermocouple terminals).

For testing under H_2_/N_2_, we have used the following protocol. First, the sample’s temperature is brought up to 140 °C. After the thermocouple reading shows a stable final temperature (~10 min), a C-V measurement is preformed to identify the structures flat-band voltage (Vfb). Once identified, the structure is held at a contact DC voltage (Vfb), and a capacitance vs. time measurement is started. N_2_ is introduced at the beginning for 10 min to validate device stability, and no change in capacitance is observed. Then, H_2_ + N_2_ with the target test concentration is inlet into the chamber, with a total flow of 500 sccm. Two mass flow controllers (MFCs) are used, one for N_2_ and another for H_2_ + N_2_ (10%); the former has a maximum flow of 1000 sccm and the latter (Horiba Z500) has a maximum 3 sccm flow; it has an accuracy of as low as 1% of the setpoint (0.03 sccm) [27]. The last step is stopping the inlet gas flow and allowing the sensor to recover.

## 3. Results

In this section, the p-Si/Al_2_O_3_/Ti/Pt structure will be characterized as a sensing device. The response to H_2_, the recovery, and the impact of the operating conditions are discussed.

### 3.1. Sensing Mechanism; Device Response and Recovery

**Figure 5:**
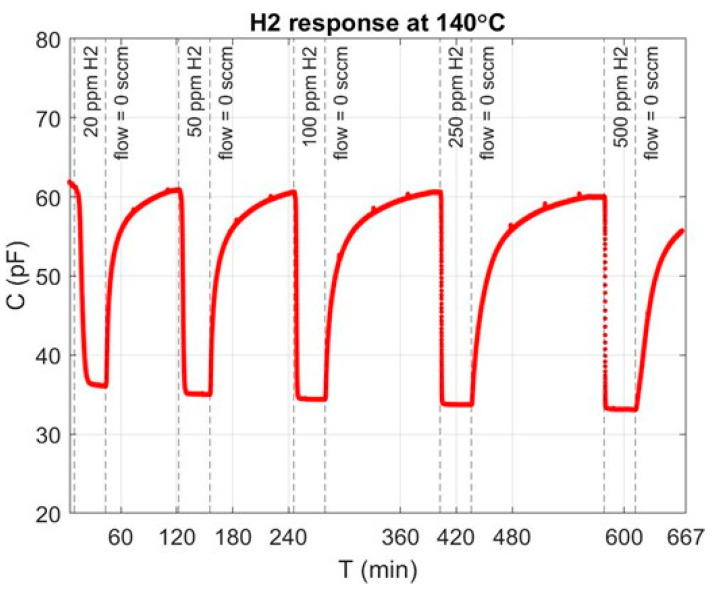
Full recovery after device exposure to different hydrogen concentrations at 140 °C with a total flow of 500 sccm.

In order to operate the MOS device under the flatband condition, a continuous DC bias of 3.3 V was applied to the Pt-gate. Capacitance time (C(t)) was determined using a Keithley 4200 SCS. In order to test the response and recovery behaviour, pulses of H_2_ were injected into the chamber at different concentrations, as shown in Figure 5. The device was held at 140 °C with a constant total (N_2_ + H_2_) inlet gas flow of 500 sccm. At a concentration of 20 ppm, the capacitance (∆C) varies by approximately 25 pF. When there is no gas flowing into the chamber (0 sccm inlet H_2_ flow), the capacitance reading reverts to its initial capacitance value (61 pF), demonstrating a full recovery regime. While the falling edge response is more abrupt for higher concentrations, the recovery time is longer. This shows that the device response is directly dependent on the H_2_ concentration. In Figure 5, the capacitance is the parameter of choice to show the MOS sensor response to hydrogen; in reality, the interface potential is what effectively changes due to the hydrogen atoms adsorption at the metal–insulator interface (Pt/TiO_x_/Al_2_O_3_). The potential at the interface changes according to ΔV = μ*ni/ε_0_. As shown above, our MOS structure, as expected, has three operating regimes: (1) accumulation (low voltage values), where the capacitance saturates to higher values; (2) depletion, where the capacitance decreases as a function of the gate voltage; (3) inversion, where the Si channel has inverted, and minority carriers are attracted to the semiconductor/oxide interface. The voltage at the gate is set to 3.3 V to keep the MOS structure at the sensitive operating point on the C-V curve and maximize the sensitivity and change in capacitance with respect to change in voltage. If the voltage is too high/low, then no change in capacitance would be observed. 

Figure 5 also shows the regeneration behaviour, since the capacitance returns to its initial value at 61 pF. It is worth noting that the recovery time is longer than the response time, as the H-H recombination reaction is slower than the dissociation reaction rate [12]. The cyclic hydrogen exposure test demonstrates the reversible nature of the sensing mechanism that is essential for any sensor.

### 3.2. The 90% Response Time

**Figure 6:**
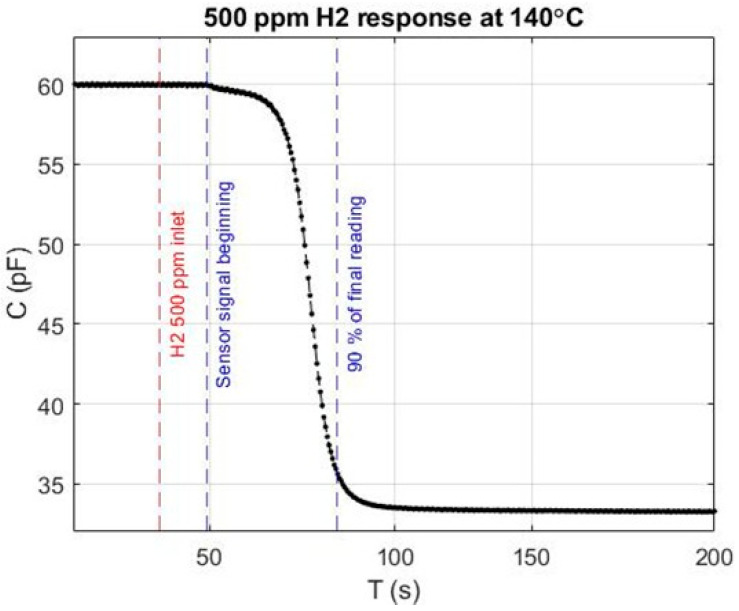
Response at 500 ppm hydrogen concentration at 140 °C with a total flow of 500 sccm.

For a given concentration, the C(t) curve can be used to estimate the 90% response time, commonly used to characterise the device performance [28]. The response curve shown in Figure 6 depends on multiple factors, such as the chamber volume, its geometry, and the gas flow rate. The sensor’s total response comprises intrinsic as well as extrinsic response time components. Some factors have less impact than others; the diffusion time though the 100 nm thick Pt gate is one example. The diffusion coefficient of hydrogen gas through Pt at 30 °C is 2.3 × 10^−13^ m^2^/s [29,30]; we can use this equation to estimate the diffusion time, (t = x^2^/2D), t (diffusion time) = (100 × 10^−9^)^2^/(2 × (2.3 × 10^−13^)) = 22 ms. Here, x is the Pt thickness in m and D is the diffusion coefficient (m^2^/s). At 140 °C, the operating temperature of the sensor, the diffusion time is even lower than 22 ms. Other factors also may be proprietary to the structure itself. 

In Figure 6, the red dashed line marks the moment at which hydrogen starts to flow into the chamber (t_H2 on_), and the blue dashed line marks the time at which the device reaches 90% of its final reading. The time elapsed between those two moments defines the response time (t_90%_) in the context of this study. For example, for 500 ppm using the 90% response time method, the response time is 56 s. It should be noted that this work aims to demonstrate the use of an Al_2_O_3_-MOS for hydrogen sensing applications. A shorter response time is certainly attainable testing is conducted in a smaller chamber.

### 3.3. Capacitance Variation (∆C) and Response Time (t90%)

**Figure 7:**
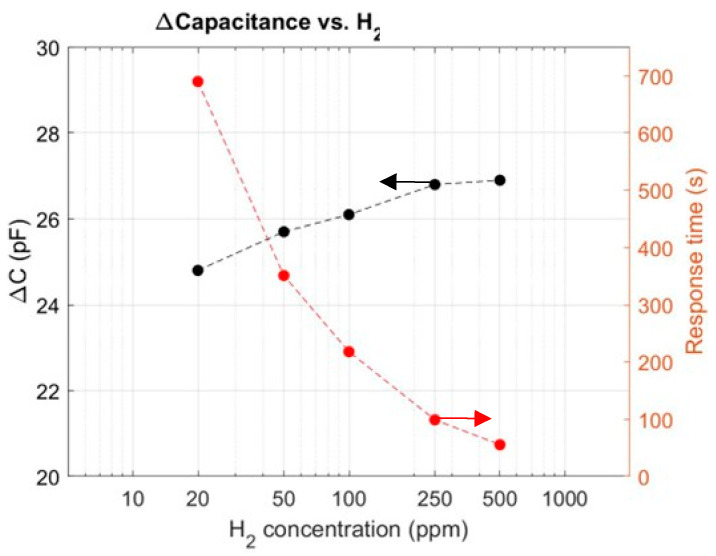
Capacitance variation and response time as a function of hydrogen concentration at 140 °C at a total gas flow of 500 sccm.

The capacitance variation (∆C), defined as the change before and after H_2_ injection, has been shown to be dependent on the H_2_ concentration; meanwhile, t_90%_ can be used to characterise how fast the device’s response is. These two parameters can be used to characterise the MOS sensor response and they are plotted in Figure 7. Note that every data point is the average of 1000 measured data points, with a standard deviation of 0.04 pF, which is 0.2% of the capacitance variation to 20 ppm (the lowest concentration).

In Figure 7, the response on the left-hand *y*-axis shows an increase as a function of the concentration; in contrast, on the right-hand *y*-axis, the response time varies significantly. The latter decreases from 690 s, at a concentration of 20 ppm H_2_, to approximately 56 s, at a concentration of 500 ppm. It should be noted that the lowest tested concentration was 20 ppm, since that is the lower limit of the testing station used in this study. 

Since the response (∆C) remains quite large at 20 ppm, this sensor is probably capable of detecting much lower concentrations. The lowest detection limit and the detection range are both properties inherent to the device and they mainly depend on the nature of the Pt/Ti/Al_2_O_3_ interface [10]. The response to hydrogen concentration is expected to be a nonlinear response, as demonstrated by Salomonsson et al. [9]. The MOS sensor response rather follows a Temkin isotherm, and the sensitivity is nonlinear and depends on the available/occupied hydrogen adsorption sites at the Pt-TiOx-Al_2_O_3_ interface. State-of-the-art sensors, using the closest stack [10] (p-Si/SiO_2_/Al_2_O_3_/Pt/Pd), have shown a lowest detection limit at 20 ppm [10]. Given that the results in Figure 7 are limited by the testing setup (minimum H_2_ concentration 20 ppm), the 25 pF capacitance variation at 20 ppm is on par with state-of-the-art results. The results in Figure 7 suggest that the fabricated sensor is more suitable for low-concentration detection applications. According to Boon et al. [2], for stationary H_2_-sensing applications, the required lower detection is below 100 ppm, which is well above the device’s lowest limit. Therefore, this sensor technology would be suitable for stationary applications. For automotive applications [2], where a detection range of 0–40,000 ppm and a response time of <3 s are required, further high-concentration tests should be carried out to confirm the compatibility of our MOS sensor.

### 3.4. Impact of Temperature on (∆C) and (t90%)

**Figure 8:**
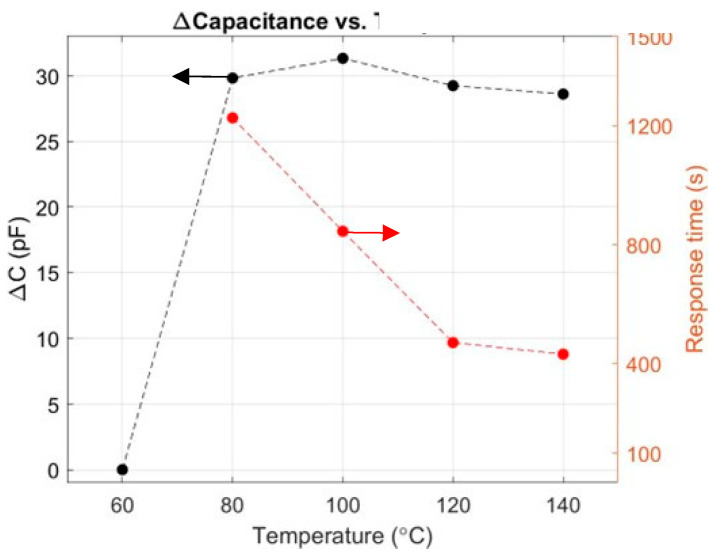
The device response to 250 ppm and response time at different temperatures at a constant total gas flow of 100 sccm.

The impact of the operating temperature on the response of Pt/Ti/ALD-Al_2_O_3_/p-Si MOS has been studied in the range 60–140 °C, as shown in Figure 8. The results show little-to-no variation in ∆C as a function of temperature. It is worth noting that, at 60 °C, the device showed no response. At such low temperatures, the limiting process is the dissociative adsorption at the Pt surface, as hydrogen diffusion is very fast through the 100 nm thick Pt film [9]. 

The sensor starts to respond at 80 °C and the capacitance variation (∆C) remains constant, even at higher temperatures. This confirms that, at a fixed concentration, the ∆C response does not vary with temperature. On the other hand, the response time significantly decreased at higher temperatures, varying from 1226 s at 80 °C to 471 s at 120 °C. The latter can be explained by a higher hydrogen dissociative adsorption reaction rate at higher temperatures [9]. Due to the testing setup, as well as sample packaging limitations, the sample has been tested up to 140 °C. Future tests in a modified setup shall enable testing up to 200 °C; they will also allow us to locate the optimal temperature and identify the sensor’s limitations/tolerance. 

### 3.5. Impact of Total Gas Flow on (∆C) and (t90%)

**Figure 9:**
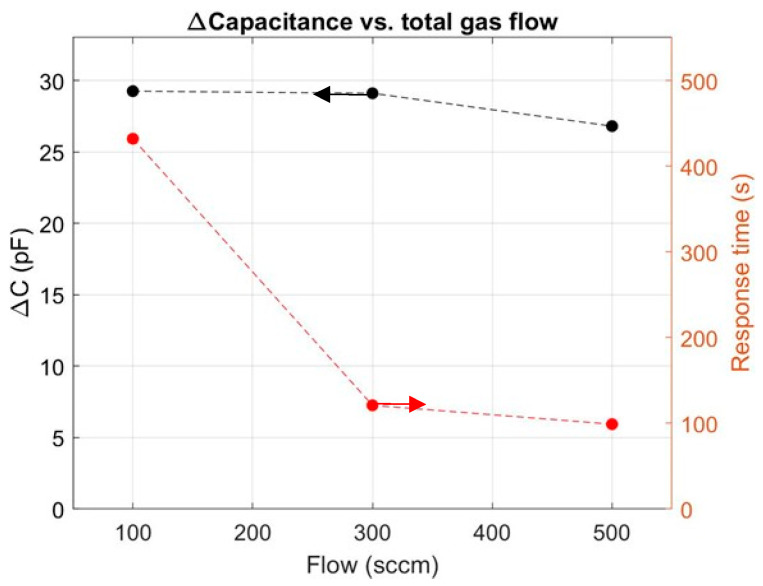
∆C and response time as functions of gas flow rate for hydrogen concentration of 250 ppm at 140 °C.

The time taken to reach a given concentration of hydrogen inside the chamber depends on the chamber volume, the total inlet gas flow (in sccm), and other factors. In Figure 9, the response, ∆C, and the response time, t_90%_, are plotted as functions of the total gas flow. Three different tests were performed, at 100, 300, and 500 sccm, and the hydrogen concentration was kept constant at 250 ppm. At a gas flow rate of 100 sccm, the response time is 432 s; at 500 sccm, it drops to 99 s. 

The decrease in response time is mostly due to the rapid increase in the chamber’s partial pressure, leading to faster hydrogen molecule transport to the platinum MOS surface. This time represents a significant portion of the extrinsic response time component.

## 4. Discussion

Multiple reports have discussed characterisation methods and equipment-dependent responses in this field [4,5,24]. Hubert et al. [4] defined a flow- and chamber-volume-dependent time constant (*τ*). The latter is used to describe the testing setup’s transient behaviour, where *τ* is the ratio between the chamber volume and the gas flow rate. The chamber volume of 160 mL used in this study corresponds to a calculated time constant *τ* of 19.2 s for a 500 sccm total flow. Ideally, sensors would be tested in the smallest volume chamber possible, to reduce the constant *τ*. For example [4], a time constant below 1 s has been calculated for a chamber volume of <3 mL, with a total gas flow of 1000 sccm.

To put this into perspective, the response time, chamber volume, and the total gas flows of different MOS- and MOSFET-based hydrogen sensors are shown in Table 1. Even though conditions differed between the experiments reported in the literature, a qualitative comparison suggests that the performance of the device presented in this study can be considered promising: (i) The obtained response time of 56 s is shorter than that demonstrated in other studies, which use a smaller chamber volume [1] (approximately two orders of magnitude higher in this study) and a higher hydrogen concentration [1] (higher by two orders of magnitude). (ii) Using a volume chamber comparable to that used by Hubert et al. [4], we attained a similar time constant τ (0.2 s); additionally, the response time of our MOS device could be further decreased and potentially brought closer to the state of the art. 

This response time remains comparable to other devices that use a similar sensing mechanism (i.e., the Pt–insulator interface in an MOS or MOSFET), even though the fabricated Pt/Ti/Al_2_O_3_/p-Si devices were tested in a chamber that was about 50 times larger (160 mL vs. 3 mL), using a gas flow that was twice as low (500 sccm vs. 1000 sccm), detecting 20-times-lower concentrations (500 ppm vs. 10,000 ppm). The response time would be reduced and potentially be closer to the reported state-of-the-art results [4] (2.3 s in Table 1).

## 5. Conclusions

This work demonstrates the use of a novel Pt/Ti/ALD-Al_2_O_3_/p-Si MOS device for hydrogen sensing applications. One key advantage is that the fabrication uses low-temperature deposition processes, such as ALD and e-beam evaporation, enabling them to be compatible with BEOL integration into CMOS transistor technologies. One transistor technology that would be suitable for such integration is the fully depleted silicon on insulator transistor, since it has a buried oxide layer that separates the bulk from the silicon channel and offers lower short-channel effects and current leakage at 140 °C [33,34]. Additionally, FDSOI transistors can withstand BEOL processing up to 550 °C [34].

MOS capacitors are fabricated using Al_2_O_3_ as a gate dielectric and Pt as a catalytic metal gate. C-V measurements have shown typical MOS behaviour with stable interface properties at room temperature. At the typical sensor’s operating temperature of 140 °C, a drift may be observed, depending on the post-oxide annealing process. This work has shown that annealing in N_2_/H_2_ eliminates this drift, enabling the use of the MOS device as a hydrogen sensor. MOS sensors were characterised under different conditions. The response of the surface potential of the MOS device to detect the presence of hydrogen has been proven. The recovery, which is important for a sensing device, is also shown when the hydrogen flow is stopped. The dependence of the capacitance variation and the response time on the hydrogen concentration is demonstrated. 

Additionally, the MOS device in this work shows a high response in 20 ppm of hydrogen and could potentially detect even lower H_2_ concentrations. It also shows a relatively short response time of 56 s, for a concentration of 500 ppm at 140 °C in a total flow of 500 sccm. Our sensor is not as sensitive as other technologies using Ga_2_O_3_ [35]; further device optimization is needed to reach an even lower detection limit. However, the main advantage of the ALD—Al_2_O_3_ MOS sensor is the fabrication process that is compatible with CMOS BEOL processing, which speeds up and facilitates its integration.

For the initial demonstration, a 38 nm Al_2_O_3_ film was used; thinner films can be expected to result in even higher sensitivities and a lower MOS structure threshold voltage. The latter would allow for an even faster response time [36]. Furthermore, using a thinner Pt gate film (e.g., 10 nm) could have an impact on the overall device’s performance and should be investigated [12]. The use of ALD TiO_2_ is also worth investigating as it may reduce the post metallization annealing step duration, since the Ti layer is already oxidized. Furthermore, it is expected to impact the effective Pt–insulator interface; consequently, it impacts the number of available hydrogen adsorption sites. Other future optimizations and studies shall include responses to NH_3_ and CH_4_, and address the potential of widening the application range of the presented Pt/ALD–Al_2_O_3_-based sensor. From the literature, it is expected for Pt-gated MOS devices to detect NH_3_ and CH_4_ [37,38]. However, the distinction between analytes may be possible using their different signature bond-dissociation energies: H-H = 436 kJ/mol, H-CH = 452 kJ/mol, H-NH = 377 kJ/mol [37,38]. Finally, future testing using a modified setup that facilitates lower concentrations (1–10 ppm and below) inside a smaller chamber (<160 mL) and with a higher flow rate (>500 sccm) would enable further characterisation and reduce the extrinsic response time component.

## Figures and Tables

**Table 1:** MOS and MOSFET hydrogen sensors response time t_90_ dependence on operating conditions and testing chamber.

	τ (s)	t_90_ (s)	Vol (mL)	Flow (sccm)	H_2_ ppm	T (°C)	Oxide Method
This work	19.2	56	160	500	500	140	ALD
Hubert et al. [4]	0.2	2.3	3.4	1000	10,000	-	Thermal (FEOL)
Sasago et al. [1,17]	0.36	85	3	500	10,000	115	Thermal (FEOL)
Dwivedi et al. [31]	-	18	-	-	2960	-	RF anodization
Scharnagl et al. [32]	-	10	-	-	1000	30	-

## Data Availability

All data needed to evaluate the conclusions in the paper are present in the paper.
